# Saliva as an alternative sample type for detection of pneumococcal carriage in young children

**DOI:** 10.1099/mic.0.001394

**Published:** 2023-10-11

**Authors:** Anne L. Wyllie, Nynke Y. Rots, Alienke J. Wijmenga-Monsuur, Marlies A. van Houten, Elisabeth A. M. Sanders, Krzysztof Trzciński

**Affiliations:** ^1^​ Paediatric Immunology and Infectious Diseases, Wilhelmina Children’s Hospital, University Medical Center Utrecht, Utrecht, The Netherlands; ^2^​ Epidemiology of Microbial Diseases, Yale School of Public Health, New Haven, Connecticut, USA; ^3^​ Centre for Infectious Disease Control, National Institute for Public Health and the Environment (RIVM), Bilthoven, The Netherlands; ^4^​ Spaarne Gasthuis Academie, Spaarne Gasthuis, Hoofddorp, The Netherlands

**Keywords:** *Streptococcus pneumoniae*, streptococci, carriage, molecular diagnostics, saliva

## Abstract

For children, the gold standard for the detection of pneumococcal carriage is conventional culture of a nasopharyngeal swab. Saliva, however, has a history as one of the most sensitive methods for surveillance of pneumococcal colonization and has recently been shown to improve carriage detection in older age groups. Here, we compared the sensitivity of paired nasopharyngeal and saliva samples from PCV7-vaccinated 24-month-old children for pneumococcal carriage detection using conventional and molecular detection methods. Nasopharyngeal and saliva samples were collected from 288 24-month-old children during the autumn/winter, 2012/2013. All samples were first processed by conventional diagnostic culture. Next, DNA extracted from all plate growth was tested by qPCR for the presence of the pneumococcal genes *piaB* and *lytA* and a subset of serotypes. By culture, 161/288 (60 %) nasopharyngeal swabs tested positive for pneumococcus, but detection was not possible from saliva due to abundant polymicrobial growth on culture plates. By qPCR, 155/288 (54 %) culture-enriched saliva samples and 187/288 (65 %) nasopharyngeal swabs tested positive. Altogether, 219/288 (76 %) infants tested positive for pneumococcus, with qPCR-based carriage detection of culture-enriched nasopharyngeal swabs detecting significantly more carriers compared to either conventional culture (*P*<0.001) or qPCR detection of saliva (*P*=0.002). However, 32/219 (15 %) carriers were only positive in saliva, contributing significantly to the overall number of carriers detected (*P*=0.002). While testing nasopharyngeal swabs by qPCR proved most sensitive for pneumococcal detection in infants, saliva sampling could be considered as complementary to provide additional information on carriage and serotypes that may not be detected in the nasopharynx and may be particularly useful in longitudinal studies, requiring repeated sampling of study participants.

## Introduction

The human nasopharynx is considered the primary niche of *Streptococcus pneumoniae *, with colonization occasionally progressing to pneumococcal disease. Disease manifestations include respiratory infections such as otitis media or pneumonia, and invasive pneumococcal disease (IPD) such as bacteraemic pneumonia or sepsis with or without meningitis [1]. Current vaccination strategies are targeted towards the polysaccharide capsule, which is considered the primary pneumococcal virulence factor [2]. With over 100 known capsular types (serotypes) [3], current vaccination coverage is limited to either a maximum of 20 serotypes included in the conjugated polysaccharide vaccines (PCVs) or 23 serotypes for the polysaccharide vaccine (PPSV23). PCV vaccination of children (the key transmitters of pneumococcus) not only protects against vaccine serotype (VT) disease, but also against VT carriage. Moreover, PCVs lead to herd protection in other age groups, which has led to reductions in VT disease in older adults [1, 4–12]. However, the reduction in VT carriage has been followed by an increase in non-vaccine serotype (NVT) carriage [12–14]. This serotype replacement [10] has eroded the benefits of vaccination.

For any strategy aiming to prevent pneumococcal disease, knowledge of pneumococcus reservoirs in the population is essential and surveillance of carriage can provide data on vaccine effects before any impact on disease can be observed [15]. Pneumococcal carriage detection has been instrumental in informing us on the impact of vaccination against pneumococcal disease [16, 17]. The gold standard method for carriage detection is conventional culture of a nasopharyngeal swab. Recommendations set forth by a working group convened by the World Health Organization (WHO) in 2013 reiterated this for infants and children with an additional oropharyngeal swab being recommended to improve the sensitivity of detection in adults should resources be available [18]. Historical records from the pre-antibiotic era, however, reported high carriage rates ranging between 39 and 54 % across all ages when oral (saliva) samples were tested with a sensitive animal inoculation method [19–22]. This suggested to us that sampling of the oral site might increase carriage detection [23]. In line with this, we and others have demonstrated the potential of molecular methods for increased pneumococcal detection in older age groups when oropharyngeal swabs [24–27] or saliva samples were tested alongside nasopharyngeal swabs [23, 24, 28].

Since young children are typically the focus of surveillance of pneumococcal carriage prior to or following updated vaccination strategies, we explored the sensitivity of culture and molecular (qPCR) methods for pneumococcal carriage detection in nasopharyngeal swabs and saliva samples collected from children aged 24 months to investigate whether nasopharyngeal sampling was also underdetecting carriage prevalence in this population.

## Methods

### Study design

Paired nasopharyngeal swabs and saliva samples were collected from 330 PCV7-vaccinated 24-month-old children [14, 29] in a prospective cross-sectional study conducted in the Netherlands during the autumn/winter season of 2012/2013. Detailed descriptions of the study population and primary results for pneumococcal carriage detection in the nasopharyngeal samples were reported previously [14, 29].

### Collection of saliva samples

Prior to sample collection, informed consent was obtained from both parents/caregivers. For saliva collection, a sponge lollipop (Oracol, Malvern Medical Developments, Worcester, UK) was placed in the front part of the child’s mouth for approximately 1 min until saturated with saliva [30]. The wet sponge was then placed into a sterile 5 ml syringe and the lollipop stick was withdrawn through the narrow opening, leaving only the sponge inside the syringe. Using the plunger, the sponge was compressed, and the saliva was transferred to a 2 ml cryovial prefilled with 0.1 ml of 50 % glycerol water solution (made in-house). Samples were transported to the diagnostic laboratory on dry ice and stored at −80 ˚C.

### Pneumococcal carriage and serotype detection

Testing of nasopharyngeal and saliva samples occurred during the same study period; methods used for the testing of nasopharyngeal swabs have been previously described [29]. Briefly, nasopharyngeal swabs were processed by conventional diagnostic culture for pneumococcal carriage detection with isolates serotyped using the Quellung method [31], DNA extracted from plate harvests was tested using qPCR.

Saliva samples were thawed in batches and 100 µl of saliva cultured on Columbia agar with 7 % defibrinated sheep blood and gentamicin 5 mg l^−1^, a medium selective for pneumococcus*,* as previously described [14, 24]. Following overnight incubation at 37 °C and 5 % CO_2_, all growth was harvested from all culture plates into 2,1 ml brain heart infusion broth (Oxoid, Badhoevedorp, the Netherlands) supplemented with 10 % (v/v) glycerol and stored frozen at −80 °C [23]. These samples were considered to be culture-enriched for pneumococcus. DNA was extracted from 200 µl of culture-enriched saliva samples as previously described [23] and then tested using qPCR for the presence of two pneumococcal-specific genes, *piaB* [25, 29] and *lytA*.[32]. Samples were classified as positive for *S. pneumoniae* when *C*_T_ values for both targeted genes were <40 [23, 24, 26, 27].

Regardless of the outcome of *piaB* and *lytA* qPCR testing, all saliva samples from children were tested using qPCR for the presence of sequences specific for pneumococcal serotypes/serogroups 1, 3, 6A/B/C/D, 7 A/F, 8, 9 A/N/V, 10A/B, 12A/B/F, 14, 15A/B/C, 19A, 20, 23F, 33 A/F/37[33], 11A/D, 16F, 18B/C and 19F [34]. Pneumococcus-positive samples were classified as positive for pneumococcal serotype/serogroup from assays determined to be specific for pneumococcus, when *C*_T_ values for targeted genes were <40.

The results from the detection of pneumococcus in saliva samples obtained in the current study were analysed together with the results of pneumococcal carriage and serotype detection in their paired nasopharyngeal swabs, which have been previously reported [29].

### Statistics

Statistical analyses were conducted using GraphPad Prism v5.0 (GraphPad Software, San Diego, CA, USA). The sensitivity of a sample type for the detection of pneumococcal carriage was determined as the number of carriers identified by that sample type (nasopharyngeal swab or saliva) and testing method (culture or qPCR) over the total number of carriers detected by all methods applied in the study. For pneumococcal serotypes detected, the frequency of carriage was calculated for each serotype by the total number of samples testing positive for that serotype by either Quellung or qPCR over the total number of pneumococcal carriers detected for each study group. If a serotype was not detected in the method being compared (nasopharyngeal swabs tested by Quellung, nasopharyngeal swabs tested by qPCR or saliva samples tested by qPCR) it was assigned a value of 0.5× the fraction representing a single carrier for that method. Differences in pneumococcal serotype carriage were evaluated using McNemar’s test and differences in serotype detection were evaluated using two-way Fisher’s exact tests. An estimate was considered statistically significant at *P*<0.05.

## Results

From the 293 24-month-old children previously reported on [29] for pneumococcal carriage detection in their nasopharyngeal samples, 288 (98 %) matching saliva samples were available for inclusion in the current study. The results summarized in Table 1 depict differences in pneumococcal carriage detection between the two sample types and detection methods (culture vs qPCR). Here, we report on the sensitivity of each method, defined as the number of individuals that tested positive for pneumococcal carriage by each method, over the total number of pneumococcal carriers detected by any of the detection method applied in the study.

**Table 1.** Sensitivity of pneumococcal carriage detection in paired nasopharyngeal and saliva samples collected from PCV7-vaccinated 24-month-old children using conventional and molecular detection methods

	24-month-olds (*n*=288) [29]	
**Detection method**	Carriage	Sensitivity
**Culture**		
Nasopharyngeal	161 (60 %)	0.74
Saliva	ND*	0
**qPCR**		
Nasopharyngeal	187 (65 %)†	0.85‡
Saliva	155 (54 %)	0.71
**Overall**§	**219** (76 %)^##^	**1**

* ND, not detectable by culture due to abundant polymicrobial growth on culture plate.

† *P*<0.05, ^##^ *P*<0.01 (McNemar’s test), significantly more carriers detected by this approach as compared to the gold standard method of culture of nasopharyngeal swab (top row).

‡Method significantly more sensitive for carriage detection (*P*<0.05) than any other tested in the particular study group.

§The total number of individuals that tested positive for pneumococcal carriage by either sample type and detection method (culture and/or qPCR).

### Pneumococcal carriage detected by culture

Carriage prevalence as detected by culture was in line with contemporary rates reported by others [14, 35, 36]. Culture-based detection of pneumococcal carriers when testing nasopharyngeal swabs was of relatively high sensitivity (0.74). However, isolation of live pneumococci from saliva at the initial culture step was not possible due to abundant polymicrobial growth on plates selective for pneumococcus [23, 24], thus the sensitivity of pneumococcal carriage detection by culturing saliva at the primary detection step was zero.

### Pneumococcal carriage detected in culture-enriched samples by qPCR

Testing culture-enriched samples by qPCR significantly increased pneumococcal detection as compared with culture detection. Testing culture-enriched nasopharyngeal samples by qPCR was the most sensitive method of carriage detection (0.85) and identified significantly more carriers than testing culture-enriched saliva samples by qPCR (187/288, 65 %, versus 155/288, 54%, respectively; *P*=0.002). Nonetheless, with 32/219 (15 %) of carriers identified only testing positive in their saliva sample, testing saliva significantly contributed to the overall carriage prevalence detected compared to testing nasopharyngeal swabs alone (219/288 versus 187/288, respectively; *P*=0.002). Importantly, there was no significant difference between the detection of carriage when testing saliva using qPCR compared with the gold standard culture of nasopharyngeal swabs (155/288 versus 161/288, respectively; *P*=0.59).

Overall, 219/288 (76 %) children were positive for pneumococcal carriage when the results from both sample types and both detection methods were combined.

### Effect of sample type and testing method on pneumococcal serotype detection

To also evaluate saliva for pneumococcal serotype detection in this young age group, we compared data generated in the current study to the previously described nasopharyngeal serotype carriage data [29], detected using either the WHO recommended culture-based approach [18] or by testing culture-enriched nasopharyngeal samples using qPCR (Table S1, available in the online version of this article).

**Fig. 1.**
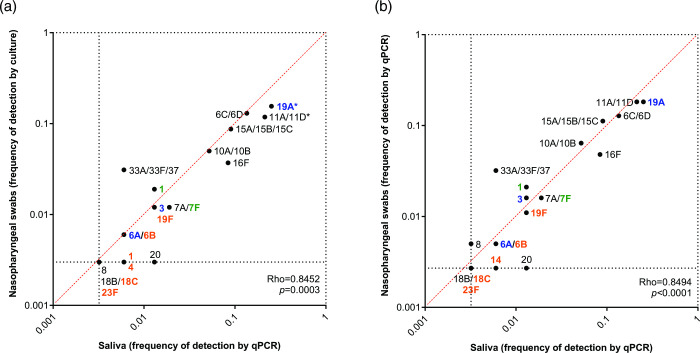
Comparison of the frequency of pneumococcal serotypes detected in nasopharyngeal versus saliva samples from 24-month-old children, when tested by conventional culture or qPCR. Graphs depict the correlation between the frequency of serotypes (from the subset targeted by qPCR) detected in nasopharyngeal swabs by (**a**) culture or (**b**) qPCR compared to detection in saliva samples using qPCR. The frequency of carriage was calculated for each serotype by the total number of samples testing positive for that serotype by either the conventional culture-based Quellung method or qPCR, over the total number of pneumococcal carriers detected for each study group. If a serotype was not detected in the method being compared (nasopharyngeal swabs tested by Quellung, nasopharyngeal swabs tested by qPCR or saliva samples tested by qPCR) it was assigned a value of 0.5× the fraction representing a single carrier for that method. Serotypes not detected by both methods were excluded from correlation calculations. Colour indicates serotypes targeted by PCV7 (orange), PCV10 (green), PCV13 (blue) or NVTs (black). Asterisks depict serotypes that differed significantly (*P*<0.05) in frequency of carriage between sample types. Black dotted lines indicate the minimum (0; represented as the value of 0.5× the fraction representing a single carrier for that method) and maximum (1) values for carriage frequency. The red dotted line indicates values of equal frequency between methods.

There was a strong correlation between the frequency of a pneumococcal serotype being detected in nasopharyngeal samples by culture and its frequency of detection in saliva by qPCR (rho=0.845; *P*<0.001). Despite fewer overall carriers being detected when testing saliva with qPCR, among carriers, we identified a higher rate of serotypes co-carried in saliva samples (33/155, 21.3 % samples positive for 2 or more serotypes; average=1.25 serotypes/sample) as compared to testing nasopharyngeal swabs by both culture (3/161, 1.9 % samples positive for 2 or more serotypes; average=1.02 serotypes/sample) and qPCR (29/187, 15.5 % samples positive for 2 or more serotypes; average=1.17 serotypes/sample). As compared to culture-based pneumococcal detection in nasopharyngeal samples, qPCR-based detection in saliva detected significantly more carriers of serotypes 11A/D (19/161, 12 %, versus 33/155, 21 %; *P*=0.033), 19A (25/161, 16 %, versus 39/155, 25 %; *P*=0.036) and PCV13 VTs overall (35/161, 22 %, versus 50/155, 32 %; *P*=0.042) (Fig. 1a). There was an even stronger correlation between the frequency of detection for a serotype when nasopharyngeal and saliva samples were both tested using qPCR (rho=0.849, *P*<0.0001). Moreover, when both sample types were tested by qPCR, there were no significant differences in the carriage frequencies of serotypes between nasopharyngeal or saliva samples (Fig. 1b).

**Fig. 2.**
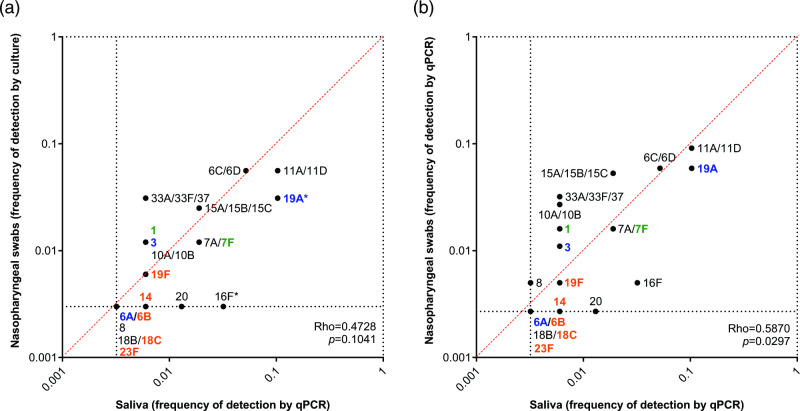
Investigating the additive effect of testing samples obtained from different sites on the detection of pneumococcal serotypes in 24-month-old children. Graphs depict serotype carriage frequency in pneumococcal carriers who tested positive for a specific serotype in only one sample type; individuals who were positive for the same serotype in both sample types were excluded from analysis. The frequency of carriage was calculated for each serotype by the total number of samples testing positive for that particular serotype by either Quellung or qPCR, over the total number of pneumococcal carriers detected by that method. If a serotype was not detected in the method being compared (nasopharyngeal swabs tested by Quellung, nasopharyngeal swabs tested by qPCR or saliva samples tested by qPCR), it was assigned a value of 0.5× the fraction representing a single carrier for that method. Serotypes not detected by both methods were excluded from correlation calculations. While (**a**) significantly higher carriage frequencies of serotypes 16F and 19A were detected when saliva was tested by qPCR as compared to the gold standard culture-based method (*P*<0.05), (**b**) no serotype was detected more frequently by either sample type when samples were tested by qPCR. Colour indicates serotypes targeted by PCV7 (orange), PCV10 (green), PCV13 (blue) or NVTs (black). Asterisks depict serotypes that differed significantly (*P*<0.05) in frequency of carriage between sample types. Black dotted lines indicate the minimum (0; represented as the value of 0.5× the fraction representing a single carrier for that method) and maximum (1) values for carriage frequency. The red dotted line indicates values of equal frequency between methods.

With similar rates of serotype detection in both nasopharyngeal and saliva samples collected from children, we were interested in the benefit of each sample type for its contribution to serotype detection. Therefore, to investigate the additive effect of samples obtained from different sites on serotype detection in children, we reanalysed the serotyping data, excluding children who tested positive for the same serotype in both their nasopharyngeal swab and saliva sample. As compared to culture detection in nasopharyngeal samples, testing saliva by qPCR detected significantly higher carriage of serotypes 16F (0/161, 0 %, versus 5/155, 3 %; *P*=0.027) and 19A (5/161, 3 %, versus 16/155, 29 %; *P*=0.012; respectively) (Fig. 2a). When both sample types were tested by qPCR, there were no significant differences in additional serotypes detected by either sample type (Fig. 2b).

## Discussion

The current culture-based recommendation for detecting pneumococcal carriage is being challenged more frequently [18, 24–26, 37–39]. While culture-independent methods improve the sensitivity of carriage detection in nasopharyngeal samples from both children [33, 40, 41] and adults [24–27, 42], we have also demonstrated improved sensitivity when these methods are applied to alternative respiratory samples from adults [24–26]. Therefore, in the current study, we conducted a direct comparison of paired nasopharyngeal swab and saliva samples collected from 24-month-old children to determine their sensitivity for pneumococcal detection when processed by culture and molecular (qPCR) methods; nasopharyngeal sampling proved superior in children.

While nasopharyngeal swabs were optimal for overall detection of pneumococcal carriage in children, saliva contributed significantly to overall carriage detection (219 total carriers detected vs 187 carriers detected by nasopharyngeal swabs alone or 155 carriers by saliva alone, *P*=0.002). For serotype detection, however, neither nasopharyngeal nor saliva samples were superior when all samples were processed by qPCR. This suggests to us that the results for serotype distribution generated using saliva are equally representative to results generated with the current gold standard method of conventional culture of nasopharyngeal swabs in children. It should be noted that culture enrichment can enhance carriage detection in low-density carriers or of secondary (or lesser) serotypes co-carried in a sample when serotype surveillance is being considered. The differences between serotypes detected in saliva compared to serotypes cultured from nasopharyngeal swabs were likely due to failure of colony picking during culture to detect co-carriage of less abundant strain(s) [43]. Importantly, overall serotype detection in saliva samples correlated well with qPCR detection in nasopharyngeal swabs. This enhanced sensitivity for both overall carriage and serotype detection are in line with findings in more recent study cohorts by Miellet *et al.*[44] and Wróbel-Pawelczyk *et al.*[45]; our observations here are not limited to one respiratory season nor geographic setting. Hence, qPCR testing of saliva samples holds potential for improving surveillance of pneumococcal carriage, providing additional insight into pneumococcal carriage compared to sampling the nasopharynx alone or providing a means to reduce the burden of study protocols through simplified sample collection. Additionally, with little-to-no discomfort from non-intrusive collection, saliva sampling is generally better tolerated compared to nasopharyngeal swabbing, encouraging greater adherence to sampling routines [24], while reducing the number of participants or samples lost due to testing aversion and refusing of sample collection [24]. This makes saliva particularly suitable for longitudinal studies.

The vast majority of epidemiological surveillance studies of pneumococcal carriage are descriptive, based on qualitative results for carriage detection, and focus on serotype distribution. They are usually solely aimed at monitoring the reduction of VTs and the emergence of NVTs. A current limitation of using qPCR to test saliva for pneumococcal carriage detection is the potential for confounding of assays due to the presence of homologous genetic sequences in closely related, non-pneumococcus streptococcal species that also inhabit the oral cavity. We provide evidence of this in the current study by ensuring that all samples that test negative for pneumococcus (i.e. qPCR-negative for *piaB* and *lytA*) are also tested in all serotyping assays. As such, it is not currently possible to specifically test for all pneumococcal serotypes in polymicrobial oral samples. Future vaccines, however, may transcend pneumococcal serotypes, with vaccines protecting against all pneumococci, independent of the capsular polysaccharide expressed. In this instance, while determining serotype-specific carriage may be only a secondary interest, accurate measures of overall pneumococcus in its ecological niche (the presence and density of all serotypes combined) will remain essential for monitoring vaccine effects and establishing study endpoints. More complex studies, with repeated sampling events, are required for better understanding of carriage dynamics (rates of acquisition and clearance, episode length) in both carriage and disease and in children versus adult populations.

With the increasing application of more sensitive detection methods, it is again being reported that, as in the early studies, pneumococcal carriage can be long lasting [46, 47], and that co-carriage of multiple serotypes is common [23, 48]. Longitudinal carriage of serotypes increases the risk of transmission, but also provides opportunities for intra- and inter-species genetic recombination [49, 50]. While findings from this and previous studies suggest to us that no single sample type should be considered to be universally superior for pneumococcal carriage detection across all age groups, collecting saliva from children – whether alone or in addition to nasopharyngeal swabs – could be considered to be a low-resource option that can relieve the burden of sample collection. Saliva is a non-invasive sample to collect that can overcome testing aversion to swabs while removing the need for healthcare workers to collect the samples [51]. While testing nasopharyngeal swabs with qPCR remained superior for overall carriage detection, testing saliva with qPCR detected a broader distribution of pneumococcal serotypes, which could partially mitigate its reduced sensitivity. If strain isolation is not of critical importance the two sample types could be merged and tested as one. Moreover, sampling saliva may have an even greater benefit in broader studies for its potential to also be tested for other upper respiratory tract commensals or pathogens [52], such as meningococci [53, 54], as well as for immune responses.

## Supplementary Data

Supplementary material 1
