# Supplementary material for: Saliva as an alternative sample type for detection of pneumococcal carriage in young children
Source: Microbiology (Reading). 2023 Oct 11;169(10):001394. doi: 10.1099/mic.0.001394 (PMC10634364; doi:10.1099/mic.0.001394)
Supplement: Supplementary material 1 [file mic-169-1394-s001.pdf]

Supplementary Table S1. Number of 24-month-old pneumococcal carriers classified as positive for serotype when nasopharyngeal and saliva samples were tested by conventional culture and/or the qPCR.

	**Sample type**						
	**Nasopharyngeal**			**Saliva**	**Overall (n=219)**		
**Serotype/ serogroup**	Culture (n=161)	qPCR (n=187)	**All (n=187)**	**qPCR only (n=155)**	Culture	qPCR	**All**
**1**$^{PCV10\#}$	3[a]	4	**4**	**2**	3	3	**5**
	0.019[b]	0.021	**0.021**	**0.013**	0.014	0.014	**0.023**
**3**$^{PCV13\#}$	2	2	**3**	**2**	2	3	**4**
	0.012	0.011	**0.016**	**0.013**	0.009	0.014	**0.018**
**6A**^{PCV13}**/6B**$^{PCV10\#}$	1/0[c]	1	**1**	**1**	1	1	**1**
	0.006/0	0.005	**0.005**	**0.006**	0.005	0.005	**0.005**
**6C/6D**	21/0	24	**24**	**21**	21	33	**33**
	0.13/0	0.128	**0.128**	**0.135**	0.096	0.151	**0.151**
**7A/7F**$^{PCV10\#}$	0/2	3	**3**	**3**	2	6	**6**
	0/0.012	0.016	**0.016**	**0.019**	0.009	0.027	**0.027**
**8**$^{\#}$	0	1	**1**	**0**	0	1	**1**
	0.000	0.005	**0.005**	**0.000**	0.000	0.005	**0.005**
**9A/9N**$^{\#}$**/9V**$^{PCV7\#}$	0/1/0	NS[d]	**1**	**NS**	1	NS	**1**
	0/0.006/0	-	**0.005**	**-**	0.005	-	**0.005**
**10A**$^{\#}$**/10B**	8/0	12	**12**	**8**	8	13	**13**
	0.050/0	0.064	**0.064**	**0.052**	0.041	0.059	**0.064**
**11A**$^{\#}$**/11D**	19/0	33	**34**	**33**	19	50	**50**
	0.118/0	0.176	**0.182**	**0.213**	0.087	0.228	**0.228**
**12A/12B/12F**$^{\#}$	0	NS	**0**	**NS**	0	NS	**0**
	0.000	-	**0.000**	**-**	0.000	-	**0.000**
**14**$^{PCV7\#}$	0	0	**0**	**1**	0	1	**1**
	0.000	0.000	**0.000**	**0.006**	0.000	0.005	**0.005**
**15A/15B**$^{\#}$**/15C**	4/7/3	20	**21**	**14**	14	24	**24**
	0.025/0.043/0.019	0.107	**0.112**	**0.090**	0.064	0.110	**0.110**
**16F**	6	9	**9**	**13**	6	14	**14**
	0.037	0.048	**0.048**	**0.084**	0.027	0.064	**0.064**
**18B/18C**$^{PCV7\#}$	0	0	**0**	**0**	0	0	**0**
	0.000	0.000	**0.000**	**0.000**	0.000	0.000	**0.000**
**19A**$^{PCV13\#}$	25	32	**34**	**39**	25	50	**50**

	0.155	0.171	**0.182**	**0.252**	0.114	0.228	**0.228**
**19F[PCV7#]**	2	2	**2**	**2**	2	3	**3**
	0.012	0.011	**0.011**	**0.013**	0.009	0.014	**0.014**
**20**	0	0	**0**	**2**	0	2	**2**
	0.000	0.000	**0.000**	**0.013**	0.000	0.009	**0.009**
**23F[PCV7#]**	0	0	**0**	**0**	0	0	**0**
	0.000	0.000	**0.000**	**0.000**	0.000	0.000	**0.000**
**33A/33F[#]/37**	2/2/1	6	**6**	**1**	5	7	**7**
	0.012/0.012/0.006	0.032	**0.032**	**0.006**	0.023	0.032	**0.032**
**Other[e]**	52	-	**52**	**-**	53	-	**53**
	0.323	-	**0.278**	**-**	0.237	-	**0.237**
**NT[e]**	2	-	**2**	**-**	2	-	**2**
	0.012	-	**0.011**	**-**	0.009	-	**0.009**
**Total**	163	149	**209**	**142**	164	211	**270**

[PCV7]serotype targeted by all three pneumococcal conjugate vaccines (PCVs); [PCV10]serotype targeted by PCV10 and PCV13 but not PCV7; [PCV13]serotype targeted only by PCV13.

[#]denotes serotypes also targeted by PPV23.

[a]number of carriers classified as positive for the particular serotype when tested by corresponding sample type and method.

[b]fraction of carriers positive for the particular serotype when tested by corresponding sample type and method.

[c]n/n, serotype-specific conventional culture results for serotypes indistinguishable from the serogroup when targeted by qPCR, numbers correspond to serotypes reported in the first column.

[d]NS, assay considered to be non-reliable due to lack of specificity.

[e]Serotypes detected by conventional culture only.
